# Metabolomic Profiles of the Creeping Wood Sorrel *Oxalis corniculata* in Radioactively Contaminated Fields in Fukushima: Dose-Dependent Changes in Key Metabolites

**DOI:** 10.3390/life12010115

**Published:** 2022-01-13

**Authors:** Ko Sakauchi, Wataru Taira, Joji M. Otaki

**Affiliations:** 1 The BCPH Unit of Molecular Physiology, Department of Chemistry, Biology and Marine Science, Faculty of Science, University of the Ryukyus, Okinawa 903-0213, Japan; yamatoshijimi@sm1044.skr.u-ryukyu.ac.jp (K.S.); wataira@lab.u-ryukyu.ac.jp (W.T.); 2 Research Planning Office, University of the Ryukyus, Okinawa 903-0213, Japan

**Keywords:** metabolome, LC–MS, Fukushima nuclear accident, plant physiology, radioactive pollution, *Oxalis corniculata*, creeping wood sorrel, endophytic microbe, stress response

## Abstract

The biological impacts of the Fukushima nuclear accident, in 2011, on wildlife have been studied in many organisms, including the pale grass blue butterfly and its host plant, the creeping wood sorrel *Oxalis corniculata*. Here, we performed an LC–MS-based metabolomic analysis on leaves of this plant collected in 2018 from radioactively contaminated and control localities in Fukushima, Miyagi, and Niigata prefectures, Japan. Using 7967 peaks detected by LC–MS analysis, clustering analyses showed that nine Fukushima samples and one Miyagi sample were clustered together, irrespective of radiation dose, while two Fukushima (Iitate) and two Niigata samples were not in this cluster. However, 93 peaks were significantly different (FDR < 0.05) among the three dose-dependent groups based on background, low, and high radiation dose rates. Among them, seven upregulated and 15 downregulated peaks had single annotations, and their peak intensity values were positively and negatively correlated with ground radiation dose rates, respectively. Upregulated peaks were annotated as kudinoside D (saponin), andrachcinidine (alkaloid), pyridoxal phosphate (stress-related activated vitamin B6), and four microbe-related bioactive compounds, including antibiotics. Additionally, two peaks were singularly annotated and significantly upregulated (K_1_R_1_H_1_; peptide) or downregulated (DHAP(10:0); decanoyl dihydroxyacetone phosphate) most at the low dose rates. Therefore, this plant likely responded to radioactive pollution in Fukushima by upregulating and downregulating key metabolites. Furthermore, plant-associated endophytic microbes may also have responded to pollution, suggesting their contributions to the stress response of the plant.

## 1. Introduction

Environmental pollution caused by human activities is widespread around the globe in the 21st century. Major incidents of pollution after World War II include the Great Smog in London, UK (1952) caused by particulates and gaseous mixtures, the Minamata disease outbreak in Japan (1956) caused by methylmercury, Agent Orange used during the Vietnam War (1961–1971), and the *Deepwater Horizon* oil spill accident (2010) in the Gulf of Mexico [1]. Additionally, recent human history has seen a series of pollution incidents by anthropogenic radionuclides: atomic bombs used in Hiroshima and Nagasaki, Japan (1945); atomic and hydrogen bomb experiments in Bikini Atoll (1946–1958); the Three Mile Island accident in the USA (1979); the Chernobyl nuclear accident in the Ukraine (1986); and the Fukushima nuclear accident, Japan (2011) [1]. The Fukushima nuclear accident in 2011 was the second largest nuclear accident next to the Chernobyl nuclear accident in 1986. Without question, one of the most serious environmental pollutants in this century is a group of radioactive materials released from nuclear bombs and the collapse of nuclear power plants. Today, anthropogenic ^137^Cs is detected from soil worldwide [2,3,4,5].

In the case of the Chernobyl nuclear accident, there have been inconsistencies in the biological impacts of relatively low-level radiation exposure on organisms in the surrounding environments [6,7,8,9,10]. There seem to be many reasons for these inconsistencies, but one reason may be political; the Chernobyl nuclear accident occurred in the former Soviet Union, and access to the polluted areas was limited. Another important reason may be technical. At the time of the Chernobyl nuclear accident, none of the currently available analysis technologies based on genomics, proteomics, and metabolomics had been developed. In the case of the Fukushima nuclear accident, some scientists began investigating the biological effects soon after the accident using various wild animals and plants because access to the polluted areas was not difficult from a political standpoint. There is now accumulating field-based evidence that the Fukushima nuclear accident impacted animals and plants, including birds [11,12,13], butterflies [14,15,16,17], aphids [18,19], Japanese monkeys [20,21,22], intertidal invertebrates [23], and plants [24,25,26,27,28,29], even at relatively low levels of anthropogenic radiation. However, the application of advanced technologies such as metabolomics in studies on Fukushima has not yet been sufficient.

In this study, we focused on a weed plant, the creeping wood sorrel *Oxalis corniculata*, in a contaminated field in Fukushima. This plant is the host plant of the pale grass blue butterfly, which has been used as an indicator species in Fukushima-based studies. Larvae of this butterfly eat only this plant. It has been demonstrated that the pale grass blue butterfly was impacted both genetically and physiologically by the Fukushima nuclear accident. More precisely, in view of genetic damage, the inheritance of mutation-related phenotypes over generations has been demonstrated [14,15,30,31]. In terms of physiological damage, it has been demonstrated that the ingestion of contaminated plants by butterfly larvae caused internal radiation exposure and resulted in abnormal and fatal phenotypes [14,15,32,33,34,35], although adaptive evolution to tolerate radioactive pollutants may occur over generations [36]. However, the ingestion of a ^137^Cs-containing artificial diet by larvae did not decrease survival rate, pupation rate, and eclosion rate [37]. Therefore, a positive involvement of the plant itself has been suggested to cause abnormal or fatal phenotypes in butterflies based on internal exposure experiments [38]. The plant may have experienced biochemical changes in leaves in response to radiation exposure, which has led to harmful consequences in butterflies. This field effect hypothesis is reasonable, considering that at least some plants responded to Fukushima pollution at the levels of gene expression and phenotype [24,25,26,27,28,29]. Physiological damage to butterflies is likely mediated by multiple pathways, but one of them includes biochemical plant changes in response to radiation exposure, such as changes in nutritional contents [28] and changes in secondary metabolites [29].

Plants produce secondary metabolites and proteins that are toxic to herbivorous animals such as insects. These phytotoxins include a wide variety of chemical compounds, such as cyanogens, glycoalkaloids, glucosinolates, saponins, flavones, nonprotein amino acids, furanocoumarins, condensed tannins, gossypol, protease inhibitors, lectins, and threonine dehydratase [39]. It is generally believed that herbivorous insects have evolved to cope with phytotoxins; the larvae of many species of butterflies feed on leaves containing phytotoxins such as cyanogenic glucosides and have the ability to sequester them [40]. These butterflies use these chemicals for their own defense, although many other lepidopteran insects can de novo synthesize cyanogenic glucosides [41]. The field effect hypothesis above, thus, posits that a delicate balance between phytotoxins in plants and the tolerance of phytotoxins in insects in ecosystems may have been affected by radioactive pollution.

In the present study, to examine changes in the metabolites found in *O. corniculata* in response to anthropogenic environmental radiation, we performed an LC–MS-based metabolomic analysis using plant leaf samples collected from 14 localities with various levels of ^137^Cs contamination, including Fukushima, Miyagi, and Niigata prefectures, and examined whether there were any LC–MS peaks that changed based on the ground radiation dose rate. In this way, we found candidate compounds that were upregulated or downregulated in response to different levels of radiation exposure in the plant.

## 2. Materials and Methods

### 2.1. Field Sampling

**Figure 1:**
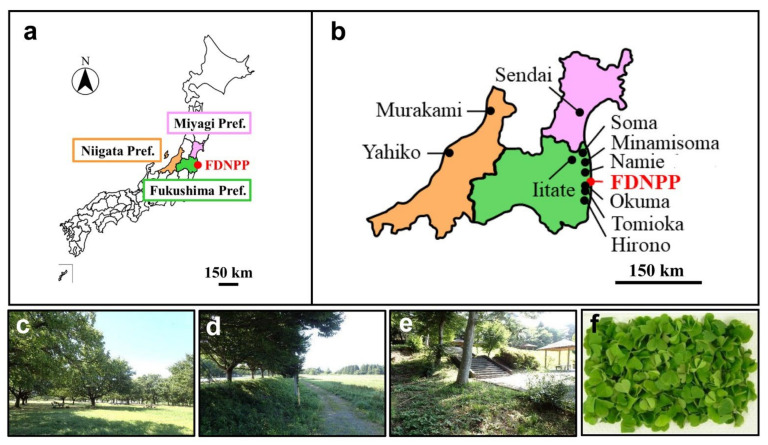
Leaf sampling: (**a**) Prefectures that include sampling localities in this study. The Fukushima Daiichi Nuclear Power Plant (FDNPP) is indicated in red; (**b**) municipalities that include 14 sampling localities in Fukushima, Miyagi, and Niigata prefectures. Minamisoma, Namie, and Iitate each have 2 or 3 independent sampling localities (see Table 1); (**c**–**e**) landscapes of sampling sites in Murakami (**c**), Minamisoma-2 (**d**), and Hirono (**e**); (**f**) Stem-separated leaves of *Oxalis corniculata* collected in Tomioka (photographed at the Kazusa DNA Institute upon sample receipt). Leaf samples from all other localities were similarly healthy when they arrived at the Institute.

We visited 14 localities in the period from 29 July 2018 to 17 September 2018 (Figure 1a–e), and two people collected leaves of the creeping wood sorrel *O. corniculata*. These localities were not affected by the tsunamis from the Great East Japan Earthquake on 11 March 2011, excluding its potential effects on the plant. Information on sampling sites and dates is listed in Table 1. Leaf samples were named OC01 to OC16 for each locality. OC05 (Minamisoma-1) and OC12 (Iwaki) were collected but were not analyzed for financial reasons.

Leaf sample collection procedures followed those described in a previous study [28]. Briefly, the plant leaves and stems were handpicked with disposable gloves so as not to damage the leaves. Leaves were further isolated from the stem. We collected leaves that were healthy and showed no signs of leaf necrosis, chlorosis, or other abnormalities. In other words, we observed no phenotypic changes under radiation stress. Leaves with damage (by insect bites, handpicking, or other unknown reasons), dead or dying leaves, leaves of different species, and other objects were eliminated manually. A minimum of 40 g of leaf samples per site was collected. Leaf samples were washed with Evian bottled natural mineral water (Evian les Bains, France).

The leaf samples (minimum of 10 g per site) were sent to the Kazusa DNA Research Institute, Kisarazu, Chiba, Japan, under refrigeration (unfrozen) conditions (0−10 °C) for LC–MS analyses. The samples arrived at the Institute within a day. At the time of arrival, leaf quality was visually checked again at the Institute; the leaves were reasonably fresh and green (Figure 1f). A portion of the leaf samples (approximately 30 g per site) was saved for an analysis of radioactivity concentration at the University of the Ryukyus.

### 2.2. Measurements of Ground Radiation Dose Rates and Radioactivity Concentrations

At the sampling sites, we measured the ground radiation dose rate (often simply called the ground dose) using a Hitachi Aloka Medical TCS-172B scintillation survey meter (Tokyo, Japan) for 90 s at 3 points in the area of leaf collection with the probe at 0 cm from the ground surface. The ground dose was measured similarly in two localities of Iitate Village (Iitate-1 and Iitate-2), one locality of Namie Town (Namie-3), and one locality of Minamisoma (Minamisoma-3) using a Polimaster handheld gamma monitor PM1710A (Minsk, Republic of Belarus). The measured values were averaged, and they are shown in Table 1.

Procedures for measuring radioactivity concentrations were described elsewhere [28]. Briefly, the radioactivity concentration of a dried leaf sample was measured using a Canberra GCW-4023 germanium semiconductor radiation detector (Meriden, CT, USA). Measurements were conducted to obtain ^137^Cs signals until the error rate became less than 5% of the measured value within 14 days of the measurement period. Otherwise, the measurements were terminated at the end of the 14th day. In that case, a measurement value was not obtained, and it was considered zero. The results were listed in Table 1.

Ground dose and radioactivity concentration were not perfectly correlated (Supplementary Results and Supplementary Discussion, Figure S1). Based on the following considerations, we decided to preferentially use ground dose values. The leaves were more likely to be subjected to external irradiation from the ground than to internal irradiation from absorbed ^137^Cs because the plant was small, the leaves were very close to the ground, and the ground radiation included complete radiation doses of various radionuclides.

### 2.3. LC–MS: Analysis, Peak Detection, Alignment, and Annotation

The procedures for LC–MS, including analysis, peak detection, alignment, and annotation, were described elsewhere [29]. Briefly, leaf samples were prepared using methanol and MonoSpin M18 columns (GL Sciences, Tokyo, Japan). Samples were analyzed using a SHIMADZU Nexera X2 high-performance liquid chromatography (HPLC) instrument (Kyoto, Japan) with an InertSustain AQ-C18 column (2.1 × 150 mm, 3 μm particle size) (GL Sciences) connected to a Thermo Fisher Scientific Q Exactive Plus high-resolution mass analyzer (Waltham, MA, USA).

The LC–MS data obtained above were converted to mzXML format using ProteoWizard (Palo Alto, CA, USA). Peak detection, determination of ionizing states, and peak alignments were performed automatically using the data analysis software PowerGetBatch developed by the Kazusa DNA Research Institute [42,43]. The exact mass values of the nonionized compounds calculated from the adducts were used to search candidate compounds against the UC2 chemical mass databases [44] (i.e., a combination of two databases, KNApSAcK [45] and the Human Metabolome Database [46,47]) with the search program MFSearcher [48]. The LC–MS results were compiled in the Microsoft Excel file “LCMS_Result Field Data KDRI” (Supplementary File S1).

### 2.4. Statistical Analysis of the Peak Area Data

The output peak area (intensity) data from LC–MS were compiled in the Microsoft Excel file “LCMS Peak Data” (Supplementary File S2). These data were subjected to statistical analyses using MetaboAnalyst 5.0 [49,50,51], as described elsewhere [29]. We performed one-way ANOVA (analysis of variance) and used FDR (false discovery rate) < 0.05 as the criterion to consider statistical significance, and the peaks that met this criterion were examined independently, after which a Student’s *t*-test was performed as necessary, using Microsoft Excel. A principal component analysis (PCA) and heatmap analysis were performed to obtain possible relationships among the samples. In the latter, the Euclidean distance and the Ward linkage method were employed for clustering. Scatter plots were made, and mathematical model fits for linear (*y* = *ax* + *b*) and logarithmic curves (*y* = *a* × *ln*(*x*) + *b*) were performed using Microsoft Excel. Correlation coefficients for linear and logarithmic curves were obtained using JSTAT (Yokohama, Japan).

## 3. Results

### 3.1. Clustering Analyses: PCA and Heatmaps

**Figure 2:**
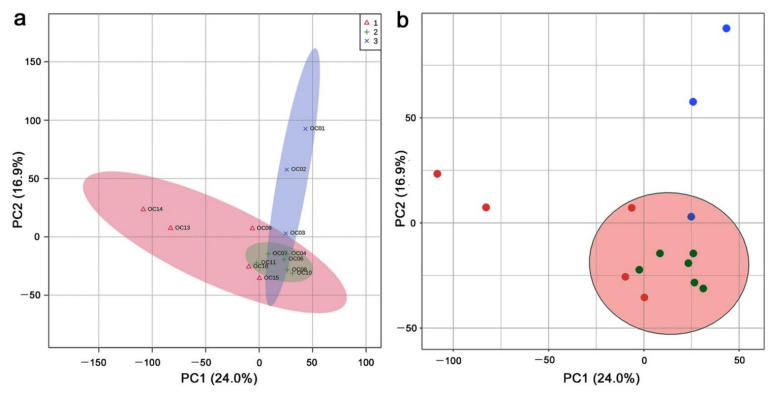
PCA using leaf samples from 14 localities in Fukushima, Miyagi, and Niigata prefectures: (**a**) Score plot, 95% confidence ranges are colored. Red, green, and blue sample dots and areas indicate high, low, and background dose-dependent groups, respectively; (**b**) K-means clustering analysis.

In the LC–MS analysis, 9554 peaks were detected, and 7967 peaks from 14 leaf samples were treated as valid peaks by MetaboAnalyst; 1587 peaks were treated as invalid because they showed a constant value across all samples or because they were detected only in one sample. To understand how these 14 samples from different localities responded to radiation, they were categorized into three groups depending on the ground radiation dose (high, low, and background levels) (Table 1). The PCA was performed using the 7967 peak area (intensity) data allocated to the 14 leaf samples (Figure 2a). PC1 and PC2 explained 24.0% and 16.9% of the variance, respectively. These percentages were not very high. The three dose-dependent groups were not well isolated from one another. Clustering by K-means (Figure 2b) and SOM (self-organizing map) (not shown) without predefining the dose-dependent groups showed that just a single group was statistically valid even when the number of groups was specified to be three. The single large group specified by K-means included nine Fukushima samples and a Miyagi sample and did not include two Fukushima (Iitate) samples (OC13 and OC14) and two Niigata samples (OC1 and OC2). This large cluster contained samples from all three dose-dependent groups.

In the PCA plot, two spots in the negative area of PC1 (OC13 and OC14) were both from Iitate Village (Fukushima Prefecture, Japan), which is located at a relatively high altitude and, thus, geologically isolated from the rest of the Fukushima localities. Another two spots in the positive area of PC2 (OC1 and OC2) were both from Niigata Prefecture, which is located on the west side of Japan (Figure 1a,b). These four samples were likely genetically or environmentally different from the rest. These results suggest that the environmental radiation dose was not a primary factor influencing peak levels in LC–MS. In other words, in terms of overall peak dynamics, the plant may not respond strongly to environmental radiation.

**Figure 3:**
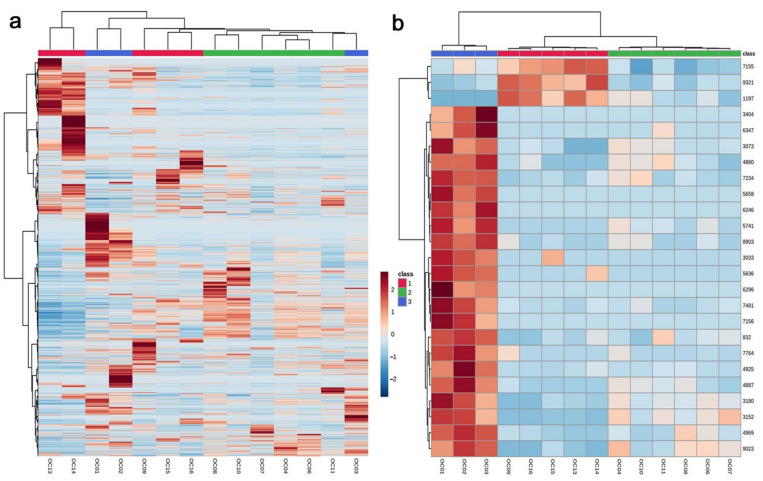
Heatmap analysis using samples from 14 localities in Fukushima, Miyagi, and Niigata prefectures. Red, green, and blue bars at the top of the heatmap indicate high, low, and background dose-dependent groups, respectively: (**a**) Heatmap using all peaks; (**b**) heatmap using the top 25 peaks.

A heatmap of all 7967 peaks also demonstrated that the three dose-dependent groups were not well justified (Figure 3a). An exception was the low-level group (shown in green bar at the top), which clustered together. However, the three groups were individually clustered when only the top 25 peaks were used to create the heatmap (Figure 3b). It appeared that a limited number of representative peaks responded to environmental radiation in a dose-dependent manner.

### 3.2. Identification of Upregulated and Downregulated Peaks

**Figure 4:**
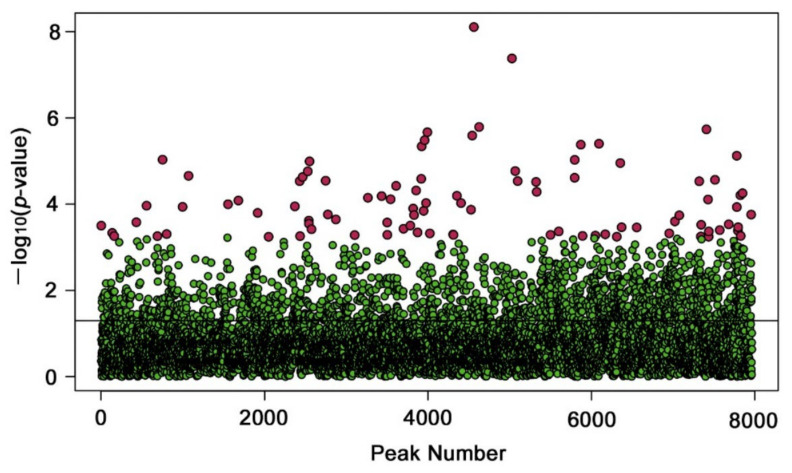
Plot of one-way ANOVA. A horizontal line at 1.301 on the *y*-axis indicates *p* = 0.05 (raw *p*-value). Red dots indicate peaks with FDR < 0.05. The peak number in the *x*-axis is adjusted for the valid number of peaks (i.e., 7967 peaks) by MetaboAnalyst.

**Figure 5:**
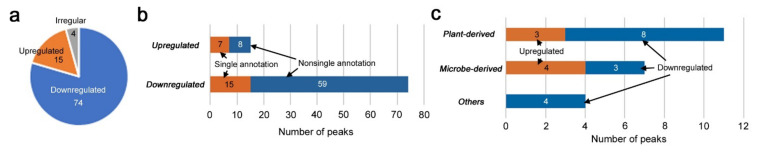
Number of upregulated and downregulated peaks of LC–MS with FDR < 0.05 (one-way ANOVA): (**a**) Pie chart; (**b**) number of upregulated and downregulated peaks with single or nonsingle annotations; (**c**) number of upregulated and downregulated peaks categorized into 3 groups, i.e., plant-derived compounds, microbe-derived compounds, and others.

Although no overall pattern justifying the three dose-dependent groups was observed in the PCA plot and heatmap using all peaks, there may have been some metabolites that were upregulated or downregulated in a dose-dependent manner. To examine this possibility, we performed one-way ANOVA with an adjusted *p*-value (FDR) cutoff at 0.05 (i.e., FDR < 0.05). We detected 93 significantly different peaks among the three groups; with FDR < 0.01, we detected 27 significantly different peaks and with FDR < 0.001, we detected two significantly different peaks (Supplementary Table S1 and Figure 4). After visual inspections of the peak values, the following numbers were obtained (Figure 5a): Among the 93 peaks, 15 peaks seemed to be dose-dependently upregulated; four peaks seemed to be upregulated (two peaks) or downregulated (two peaks) only at the low-level radiation, showing V-shaped or reversed V-shaped curves (i.e., irregular peaks); the rest (74 peaks) seemed to be downregulated.

Among the 15 upregulated peaks, seven peaks had single annotations (Figure 5b). Similarly, among the 74 downregulated peaks, 15 peaks had single annotations (Figure 5b). These peaks with single annotations were classified into three categories based on their origins: plant-derived compounds, microbe-derived compounds, and other compounds, including those of unknown or synthetic origin (Figure 5c). Plant-derived compounds were the most frequent, as expected, but unexpectedly, many microbe-derived compounds were found to be upregulated or downregulated.

### 3.3. Candidate Compounds for Upregulated and Downregulated Peaks

Upregulated peaks with single annotations (FDR < 0.05) were found, as shown in Table 2. Among them, only three upregulated peaks were considered to be plant derived. Candidate compounds for the upregulated peaks included kudinoside D, a triterpenoid saponin that has shown pharmacological activities in animal cells [52]; andrachcinidine, an alkaloid [53]; and pyridoxal phosphate, an active form of vitamin B6 that is involved in stress tolerance [54,55,56,57,58,59,60,61,62,63]. Other upregulated peaks were annotated as antibiotics, i.e., leptomycin B [64,65,66] and aldgamycin G [67], both from *Streptomyces*, a group of soil bacteria. Leptomycin B (No. 9321) was also found in the top 25 peaks for the heatmap (Figure 3b). Indeed, this peak showed the lowest FDR value (FDR = 0.0055) among the singularly annotated upregulated peaks. Additional compounds included carbamidocyclophane C, a cytotoxic compound derived from the cyanobacterium *Nostoc* sp. [68], and YM 47525, an antifungal compound of fungal origin [69].

Downregulated peaks with single annotations (FDR < 0.05) were found, as shown in Table 3. Among them, five peaks with the lowest FDR values (No. 4887, 7156, 6296, 3152, and 3073) were found in the top 25 peaks for the heatmap (Figure 3b). Candidate compounds for downregulated peaks included various types of chemicals. Plant-derived compounds were as follows: corchoionoside B (fatty acyl glucoside) [70], isoginkgetin-7-*O*-β-D-glucopyranoside (bioflavone glucoside) [71,72], sanjoinine A dialdehyde (cyclopeptide alkaloid) [73], zinolol (antioxidant) [74], acacetin-7-glucuronosyl-(1→2)-glucuronide (flavonoid) [75], tricalysioside N (*ent*-kaurane glucoside) [76], pregnadienolone-3-*O*-β-D-chacotrioside (saponin) [77], and silidianin (flavonolignan) [78]. Additionally, terreusinol, a metabolite of *Streptomyces* [79], and elloramycin E, an antibiotic from *Streptomyces* [80], were found. Trapoxin A (fungal cyclic peptide) [81,82] was also found. Other candidates included synthetic compounds [83,84,85,86,87,88]; thus, their annotations may not be accurate, although similar natural compounds may exist in the plant.

### 3.4. Correlation Analyses of Upregulated Peaks

**Figure 6:**
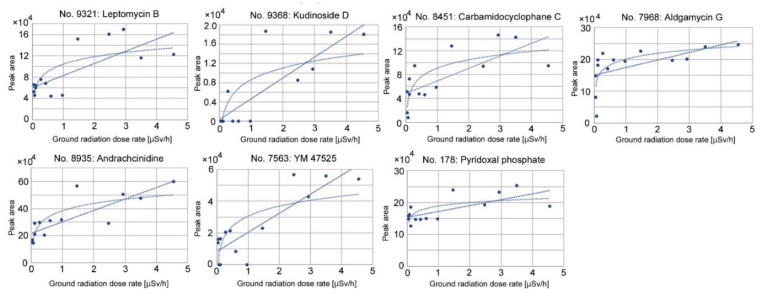
Scatter plots and linear and logarithmic fit curves of the upregulated peaks with single annotations against the ground radiation dose rate (μSv/h).

We made scatter plots and performed a correlation analysis of the upregulated peaks with single annotations to examine dose dependence (Figure 6 and Table 4). In all seven cases, correlation coefficients were reasonably high (*r* ≥ 0.69) with significantly small *p*-values. In three of seven cases, a logarithmic model fit better than a linear model, judging from correlation coefficients. Thus, these seven peaks may be upregulated in a dose-dependent manner in response to the ground radiation dose rate, confirming the ANOVA results. Correlation coefficients using the radioactivity concentration of ^137^Cs showed lower values in all cases (Table 4).

### 3.5. Correlation Analyses of Downregulated Peaks

**Figure 7:**
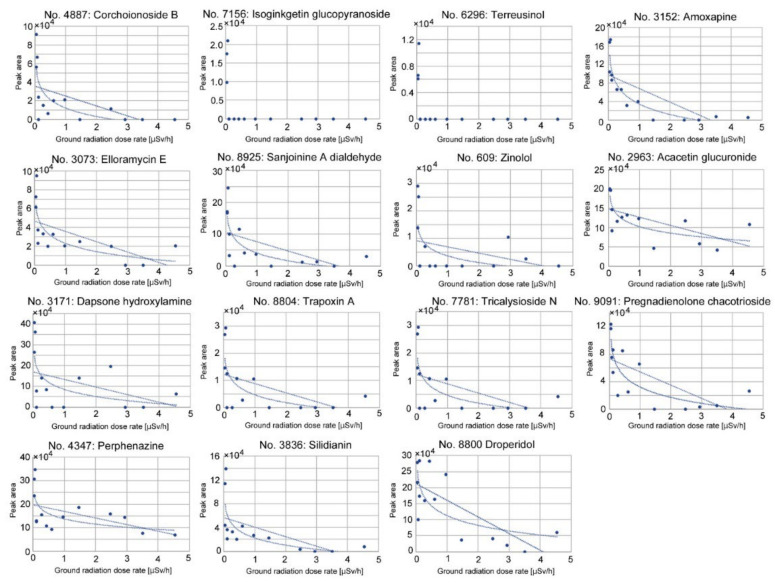
Scatter plots and linear and logarithmic fit curves of the downregulated peaks with single annotations against the ground radiation dose rate (μSv/h).

We also made scatter plots and performed a correlation analysis of the downregulated peaks with single annotations (Figure 7 and Table 5). Overall, the absolute values of coefficients were reasonably high (|*r*| ≥ 0.59), with reasonably small *p*-values. A logarithmic model fit better than a linear model, judging from correlation coefficients, in all cases examined except No. 8800, but in No. 7156 and No. 6296, peak levels were all or none. As seen in these two all-or-none cases, the downregulated response appeared to be very sensitive to the ground radiation dose rate, showing a steep logarithmic decline. It can be concluded that these 15 peaks were downregulated in a dose-dependent manner in response to the ground radiation dose rate, confirming the ANOVA results. Correlation coefficients using the radioactivity concentration of ^137^Cs showed lower absolute values in all cases (Table 5).

### 3.6. Peaks Upregulated or Downregulated at the “Low Level”

As mentioned before, among the peaks with FDR < 0.05 (ANOVA), there were four peaks that seem to be upregulated (No. 4745 and 7256) or downregulated (No. 8508 and 750) only at the “low level”. Among them, No. 4745 lacked annotation due to unknown chemical formula, and No. 7256 was singularly annotated as K_1_R_1_H_1_ (peptide), although its relevance to natural compounds in plants was unclear. Additionally, No. 8508 showed no database hit, and No. 750 was singularly annotated as DHAP(10:0), decanoyl dihydroxyacetone phosphate.

**Figure 8:**
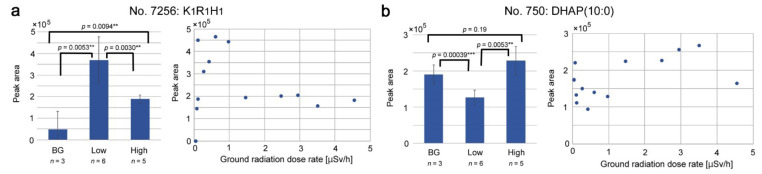
Bar graphs and scatter plots of singularly annotated peaks upregulated or downregulated at the “low level” of the ground radiation dose rate (μSv/h). Mean ± standard deviation values and results of *t*-test (raw *p*-values) are shown. Asterisks indicate levels of statistical significance; **, *p* < 0.01; ***, *p* < 0.001: (**a**) No. 7256, K_1_R_1_H_1_; (**b**) No. 750, DHAP(10:0).

We made bar graphs and scatter plots for No. 7256 and No. 750 (Figure 8). In No. 7256 (Figure 8a), the low-level group was significantly larger than the background and high-level groups in terms of peak area. As expected, correlation coefficients were low, i.e., *r* = −0.11 (*p* = 0.70) for a linear model and *r* = 0.24 (*p* = 0.41) for a logarithmic model. In contrast, in No. 750 (Figure 8b), the low-level group was significantly smaller than the background and high-level groups in terms of peak area. Unexpectedly, correlation coefficients were not very low, i.e., *r* = 0.54 (*p* = 0.048) for a linear model and *r* = 0.42 (*p* = 0.13) for a logarithmic model.

## 4. Discussion

In this study, we collected leaf samples of creeping wood sorrel, the host plant of the pale grass blue butterfly, from contaminated localities in Fukushima. These leaves had been chronically exposed to anthropogenic radiation in the field and were subjected to an LC–MS-based metabolomic analysis. Somewhat surprisingly, an overall dose-dependent trend for metabolomic changes in plants coping with radioactive environments was not observed in the PCA. One might think that this may be because the environmental pollution levels of the collection localities were not high enough for the plant to change its levels of many metabolites. However, this is not necessarily the case. In a previous study, the same plant species in Okinawa was subjected to acute external irradiation, and an overall irradiation response was clearly observed in GC–MS-based analyses despite low-level irradiation from the contaminated soil in Fukushima [29]. In contrast, such an overall response was less clear in an LC–MS-based analysis, suggesting that genetic background was a larger contributor to peak variations than external irradiation itself in a group of compounds amenable to LC–MS [29].

Notably, no identical compounds were annotated between the previous study [29] and the current study. It is also important to note that in the former study, the plant was exposed only externally, whereas in the latter study, the plant was exposed both externally and internally. Nevertheless, in both studies, compounds related to *Streptomyces* were found, i.e., three peaks in the present study and four peaks in the previous study [29]. In this sense, acute exposure under laboratory-based conditions (the previous study [29]) and chronic exposure under field conditions (the present study) may result in different outcomes in the plant but with some similarities. In the case of chronic exposure, the field plants may have already acclimated or adapted genetically to the radioactively contaminated environments by changing the levels of a relatively small number of key metabolites. 

Although an overall dose-dependent response in plant samples from Fukushima was not observed, we identified some upregulated and downregulated peaks in response to ground radiation dose. In most upregulated and downregulated peaks, logarithmic fits were better than linear fits. Such a nonlinear response may be widely seen as a plant response to low-dose radiation. An all-or-none response was also observed in downregulated peaks.

There were three upregulated peaks annotated as plant-derived compounds: kudinoside D, andrachcinidine, and pyridoxal phosphate. Kudinoside D is a type of triterpenoid saponin and is known to have biological activities [52]. Importantly, when the ground radiation dose was close to zero, kudinoside D was rarely detected, showing nearly an all-or-none response. Thus, this compound may confer high stress tolerance against environmental radiation in plants. Andrachcinidine is an alkaloid [53]. These two compounds may be stress protectants for the plant and may also function to ward off herbivorous insects. They may cause abnormal and fatal phenotypes in pale grass blue butterfly larvae.

Interestingly, pyridoxal phosphate is an activated vitamin B6 known to function in response to salt stress and other types of stress in plants [54,55,56,57,58,59,60,61]. In addition to its function as a cofactor of stress protectant enzymes, vitamin B6 functions as an antioxidant [62,63]. Notably, this compound is also known to be upregulated in response to ultraviolet irradiation in plant acclimation [61]. The present study suggests that anthropogenic environmental irradiation in Fukushima may also cause upregulation of pyridoxal phosphate to cope with radiation stress in *O. corniculata*. Furthermore, based on the existing literature [54,55,56,57,58] and the present data, upon irradiation, sodium may be expelled from the plant more efficiently to induce salt tolerance due to the upregulation of pyridoxal phosphate. This speculation is consistent with the field-effect hypothesis that the sodium content in leaves of *O. corniculata* may decrease in response to radioactive pollutants, resulting in adverse effects on larvae of the pale grass blue butterfly due to sodium deficiency [28].

The above discussion can further be fortified by referring to KEGG (Kyoto Encyclopedia for Genes and Genomes) for metabolic reactions [89,90,91]. Among the upregulated metabolites, only pyridoxal phosphate was found in KEGG. Production of pyridoxal phosphate from pyridoxamine phosphate (R00277) or pyridoxine phosphate (R00278) also produces hydrogen peroxide. Its reverse reaction, thus, scavenges hydrogen peroxide when pyridoxal phosphate is provided from a different pathway, one of which is a reaction in pyridoxal and ATP (R00174). Interestingly, in other reactions, production of pyridoxal phosphate also produces D-alanine (R01147), D-glutamate (R01580), or L-glutamate (R07456). D-Alanine and pyridoxal phosphate are products from pyridoxamine phosphate and pyruvate, an important product of glycolysis, and D-glutamate and pyridoxal phosphate are together produced from pyridoxamine phosphate and 2-oxoglutarate, a key product in the TCA cycle [92], suggesting their involvement in a stress response associated with ATP production via glycolysis and the TCA cycle. L-Glutamate and pyridoxal phosphate are produced together by a reaction of D-glyceraldehyde-3-phosphate, D-ribulose-5-phosphate, and L-glutamine, suggesting their involvement in a stress response associated with photosynthesis. These amino acids, especially those of the D-configuration, may function as signaling molecules for a stress response [93,94,95,96].

In addition, the downregulated peaks contained various compounds, including plant-derived compounds (such as antioxidants, flavonoids, and saponins) and microbe-derived compounds (such as antibiotics). We do not know why some compounds were upregulated and functionally similar compounds were downregulated, but these compounds may be produced in different metabolic pathways and may respond to radiation stress independently.

We did not detect upregulation of antioxidants in this study other than pyridoxal phosphate, but we did detect downregulation of an antioxidant, zinolol. This is somewhat surprising because antioxidants function to nullify reactive oxygen species (ROS) that are generated by irradiation [97]. This result contrasts with a previous study of acute exposure, in which a few antioxidants were upregulated [29].

Nonetheless, there was a commonality between these studies, i.e., several peaks were annotated as compounds from soil microbes, especially antibiotics from *Streptomyces*, a group of soil bacteria. In a previous study, we thought that these microbe-derived compounds were contaminations of unrelated microbes from the soil [29]. However, leaves were washed well after collection, and no trace of contamination was seen visually. Even if a small amount of soil contamination occurred, its relative weight to the leaves of *O. corniculata* would be too small to contribute to the LC–MS results. Facing the fact that microbe-derived compounds were annotated frequently, we now think that these compounds were not from contamination but from endophytic microbes inside leaves. Indeed, many endophytic bacteria have widely been known in plants [98,99,100,101] and have been isolated from *O. corniculata* [102,103]. These microbes are probably of soil origin. These results suggest that *O. corniculata* may host various bacteria and fungi from the soil in its leaves and that compounds from these bacteria and fungi may contribute to plant functions when coping with radiation stress. Such cases of stress management appear to be common among plants [104,105]. To solidify this issue, PCR-based detection and isolated culture of endophytes from leaves may be necessary.

For the downregulated metabolites excluding synthetics, only “elloramycin” and “acacetin” were found in KEGG. The former is a bacterial metabolite, whereas the latter is a part of a plant metabolite, acacetin-7-glucuronosyl-(1→2)-glucuronide. Although elloramycin E was not found in KEGG, elloramycin A was found in the “biosynthesis of type II polyketide products” pathway (rn01057), and acacetin was found in the “flavone and flavonol biosynthesis” pathway (rn00944). These two metabolites seem to be unrelated at first glance. Interestingly, however, both reactions (R10959 and R03571) use *S*-adenosyl-L-methionine as a reactant and produce *S*-adenosyl-L-homocysteine. The present finding that elloramycin E and acacetin-7-glucuronosyl-(1→2)-glucuronide were downregulated together might indicate that the plant and its endophytes share *S*-adenosyl-L-methionine, which has important multiple roles in plant metabolism and signaling including ethylene biosynthesis and stress management [106,107,108]. This discussion supports possible functions of endophytic microbes in the *Oxalis* plant under radiation stress. 

A possible function of these microbe-derived compounds may be to protect leaves from fungal infection. This may be relevant for the survival of *O. corniculata* because this plant is a small weed, and its leaves are very close to the ground. This means that leaves were placed under high humidity conditions, which may easily allow fungal infection to occur. Indeed, fungal infections on leaves have been observed in *O. corniculata* in our laboratory when humidity conditions were not well controlled. An interesting case was reported in which fungal damage to host plant leaves of a small weed, plantain, affected the relationship between the checkerspot butterfly and its host plant [109].

Additionally, we discovered K_1_R_1_H_1_ and DHAP(10:0) as candidate compounds that responded most to the low-level radiation. The biological significance of the former is unknown, but the latter seems to be biologically significant. DHAP(10:0) is a derivative of dihydroxyacetone phosphate (DHAP), which is also called glycerone phosphate. DHAP is a metabolite in glycolysis and in the Calvin cycle. In the latter, DHAP is used to regenerate ribulose-1,5-bisphosphate, a key metabolite in the Calvin cycle. Importantly, DHAP is used for synthesis of vitamin B6 in plants but not in bacteria [110]. Thus, it is likely that the upregulation of pyridoxal phosphate detected in the present study occurred in plant cells. DHAP can be converted to glycerol-3-phosphate, which is known as a defense signaling molecule for systemic immunity in plants [111,112]. DHAP also produces methylglyoxal, a signaling molecule for abiotic stress in plants [113].

In the case of the pale grass blue butterfly in Fukushima, the high sensitivity of larvae to pollutants from the accident in the field is likely mediated by the physiological response of the host plant to the pollutants. The current study further suggested the involvement of endophytic soil microbes associated with the host plant. In the case of the monarch butterfly, larval sensitivity to neonicotinoid insecticides seems to be influenced by which host plant species larvae feed on [114]. These cases imply that the biological effects of any pollutants should be evaluated in the context of ecological interactions among plants, animals, and microbes.

## 5. Conclusions

In this study, we showed that the creeping wood sorrel likely responded to nuclear pollution in Fukushima by changing its levels of a limited number of key metabolites in a dose-dependent manner. The dose-dependent upregulated metabolites included not only plant-derived compounds (i.e., kudinoside D, andrachcinidine, and pyridoxal phosphate) but also microbe-derived compounds, some of which were antibiotics from *Streptomyces*. Pyridoxal phosphate is a stress-responding vitamer of vitamin B6 that may regulate leaf physiology, such as sodium contents. Other upregulated plant-derived compounds may function to ward off herbivorous animals, such as larvae of the pale grass blue butterfly. DHAP(10:0) is unique in that it was downregulated at the low-level radiation. DHAP(10:0) is a derivative of DHAP, which can produce vitamin B6 and stress signaling molecules. Microbe-derived compounds may also contribute to the stress response of the plant. Together, the contributions of these compounds (and their related microbes such as *Streptomyces*) to the radiation stress response should be investigated in the future and may demonstrate the importance of ecological field effects in understanding the biological impacts of the Fukushima nuclear accident. Other types of field effects [38,115,116,117] should also be investigated to understand the whole picture of the biological effects of the Fukushima nuclear accident.

## Figures and Tables

**Table 1:** Sampling information for the leaf samples, ground radiation dose rates, and radioactivity concentrations.

Sample Name	Sampling Locality	Date (2018)	Ground Dose Rate [μSv/h] (*1)	^137^Cs Radioactivity Concentration [Bq/kg]
**OC01**	Murakami City, Niigata Pref.	29 Jul	0.07 (B)	0
**OC02**	Yahiko Village, Niigata Pref.	30 Jul	0.04 (B)	0
**OC03**	Sendai City, Miyagi Pref.	31 Jul	0.04 (B)	4.54
**OC04**	Soma City, Fukushima Pref.	31 Jul	0.10 (L)	74.45
**OC06**	Minamisoma City, Fukushima Pref. (Minamisoma-2)	31 Jul	0.42 (L)	84.27
**OC07**	Hirono Town, Fukushima Pref.	1 Aug	0.11 (L)	7.96
**OC08**	Namie Town, Fukushima Pref. (Namie-1)	1 Aug	0.97 (L)	64.10
**OC09**	Namie Town, Fukushima Pref. (Namie-2)	1 Aug	2.45 (H)	551.16
**OC10**	Okuma Town, Fukushima Pref.	1 Aug	0.60 (L)	424.45
**OC11**	Tomioka Town, Fukushima Pref.	1 Aug	0.27 (L)	135.98
**OC13**	Iitate Village, Fukushima Pref. (Iitate-1)	17 Sep	3.50 (H)	213.72
**OC14**	Iitate Village, Fukushima Pref. (Iitate-2)	17 Sep	2.94 (H)	494.74
**OC15**	Namie Town, Fukushima Pref. (Namie-3)	17 Sep	4.55 (H)	717.65
**OC16**	Minamisoma City, Fukushima Pref. (Minamisoma-3)	17 Sep	1.46 (H)	175.23

(*1) Three groups were set depending on the relative levels of ground radiation dose rate (*R*): H (high, *R* ≥ 1.00); L (low, 0.10 ≤ *R* < 1.00); and B (background, *R* < 0.10). Samples OC05 (Minamisoma-1) and OC12 (Iwaki) were collected but not analyzed.

**Table 2:** Summary of the upregulated peaks with single annotations.

No.	Formula	Exact Mass	Annotation (Compound Name)	Possible Function	Origin
**9321**	C_33_H_48_O_6_	540.345	Antibiotic Cl 940, Antibiotic CL 1957A, Cl 940, Elactocin, Leptomycin B, Mantuamycin	Antibiotics	*Streptomyces* sp. (Microbe-derived)
**9368**	C_47_H_72_O_17_	908.477	Kudinoside D	Triterpenoid saponin; Adipogenesis suppressor	*Ilex kudingucha* (Plant-derived)
**8451**	C_38_H_56_O_8_N_2_Cl_2_	738.341	Carbamidocyclophane C, (+)-Carbamidocyclophane C	Cytotoxic compound	*Nostoc* sp. (Cyanobacteria) (Microbe-derived)
**7968**	C_37_H_56_O_15_	740.362	Aldgamycin G	Antibiotics	*Streptomyces* sp. (Microbe-derived)
**8935**	C_13_H_25_O_2_N	227.189	Andrachcinidine, (-)-Andrachcinidine	Alkaloid	*Andrachne aspera* (Plant-derived)
**7563**	C_33_H_46_O_11_	618.304	YM 47525	Trichothecene; Fungicide	Fungus (Microbe-derived)
**178**	C_8_H_10_O_6_NP	247.025	Pyridoxal phosphate	Activated vitamin B6	Plants and microbes (Plant-derived) (*1)

Note: This table lists candidate compounds in order of smaller FDR values. Raw *p*-values and FDR values for these peaks are listed in Supplementary Table S1. (*1) There is a possibility that No. 178 was derived from endophytic microbes because they also synthesize this compound (but see Section 4. Discussion).

**Table 3:** Summary of the downregulated peaks with single annotations.

No.	Formula	Exact Mass	Annotation (Compound Name)	Possible Function	Origin
**4887**	C_19_H_28_O_9_	400.173	Corchoionoside B	Fatty acyl glucoside; Membrane stabilizer	*Corchorus olitorius* (Plant-derived)
**7156**	C_38_H_32_O_15_	728.174	Isoginkgetin-7-*O*-β-D-glucopyranoside	Bioflavone glucoside	*Ginkgo biloba* (Plant-derived)
**6296**	C_18_H_22_O_5_N_2_	346.153	Terreusinol, (+)-Terreusinol	Antibiotics	*Streptomyces* sp. (Microbe-derived)
**3152**	C_17_H_16_ON_3_Cl	313.098	Amoxapine	GPCR (G-protein-coupled receptor) inhibitor	(Others, synthetic)
**3073**	C_32_H_34_O_15_	658.190	Elloramycin E	Antibiotics	*Streptomyces* sp. (Microbe-derived)
**8925**	C_31_H_42_O_6_N_4_	566.310	Sanjoinine A dialdehyde	Alkaloid (Cyclopeptide)	*Zizyphus lotus* (Plant-derived) (*1)
**609**	C_14_H_21_O_8_N	331.127	Zinolol	Antioxidant	*Anagallis onellin* (Plant-derived)
**2963**	C_28_H_28_O_17_	636.133	Acacetin-7-glucuronosyl-(1→2)-glucuronide	Flavonoid	*Clerodendron trichotomum* (Plant-derived)
**3171**	C_12_H_12_O_3_N_2_S	264.057	Dapsone hydroxylamine	Dermatologically used drug	(Others, synthetic)
**8804**	C_34_H_42_O_6_N_4_	602.310	RF 1023A, Trapoxin A	Cyclic peptide; histone deacetylase inhibitor	*Helicoma ambiens* RF-1023 (Fungus) (Microbe-derived)
**7781**	C_28_H_46_O_11_	558.304	Tricalysioside N, (-)-Tricalysioside N	*Ent*-kaurane glucoside	*Tricalysia dubia* (Plant-derived)
**9091**	C_39_H_60_O_15_	768.393	Pregnadienolone-3-*O*-β-D-chacotrioside	Saponin	*Dioscorea panthaica* (Plant-derived)
**4347**	C_21_H_26_ON_3_SCl	403.149	Perphenazine	Dopamine receptor D2 antagonist	(Others, synthetic)
**3836**	C_25_H_24_O_10_	484.137	Silidianin	Flavonolignan	*Silybum marianum* (Plant-derived)
**8800**	C_22_H_22_O_2_N_3_F	379.170	Droperidol	Dopamine receptor antagonist	(Others, synthetic)

Note: This table lists candidate compounds in order of smaller FDR values. Raw *p*-values and FDR values for these peaks are listed in Supplementary Table S1. (*1) Sanjoinine A is a natural compound but sanjoinine A dialdehyde is a synthetically derived compound from sanjoinine A [73].

**Table 4:** Correlation coefficient *r* and its associated *p*-value of the upregulated peaks with single annotations.

No.	Brief Annotation	Ground Radiation Dose Rate [μSv/h]	Radioactivity Concentration of ^137^Cs [Bq/kg]
**9321**	Leptomycin B	*r* = 0.75, *p* = 0.0021 ** (linear)	*r* = 0.66, *p* = 0.0100 * (linear)
**9368**	Kudinoside D	*r* = 0.84, *p* = 0.0002 *** (linear)	*r* = 0.59, *p* = 0.027 * (linear)
**8451**	Carbamidocyclophane C	*r* = 0.72, *p* = 0.0037 ** (linear) *r* = 0.78, *p* = 0.0009 *** (logarithmic)	*r* = 0.51, *p* = 0.062 (linear)
**7968**	Aldgamycin G	*r* = 0.58, *p* = 0.030 * (linear) *r* = 0.72, *p* = 0.0037 ** (logarithmic)	*r* = 0.53, *p* = 0.051 (linear)
**8935**	Andrachcinidine	*r* = 0.83, *p* = 0.003 *** (linear) *r* = 0.84, *p* = 0.0001 *** (logarithmic)	*r* = 0.66, *p* = 0.0097 ** (linear)
**7563**	YM 47525	*r* = 0.87, *p* < 0.0001 *** (linear)	*r* = 0.72, *p* = 0.0035 ** (linear)
**178**	Pyridoxal phosphate	*r* = 0.69, *p* = 0.0088 ** (linear)	*r* = 0.42, *p* = 0.14 (linear)

Note: When the coefficient was better in a logarithmic model in terms of *r* than in a linear model, both are shown. If not, only the coefficient of a linear model is shown. Asterisks indicate levels of statistical significance. *, *p* < 0.05; **, *p* < 0.01; ***, *p* < 0.001.

**Table 5:** Correlation coefficient *r* and its associated *p*-value of the downregulated peaks with single annotations.

No.	Brief Annotation	Ground Radiation Dose Rate [μSv/h]	Radioactivity Concentration of ^137^Cs [Bq/kg]
**4887**	Corchoionoside B	*r* = −0.55, *p* = 0.041 * (linear) *r* = −0.76, *p* = 0.0017 ** (logarithmic)	*r* = −0.51, *p* = 0.065 (linear)
**7156**	Isoginkgetin-7-*O*-β-D- glucopyranoside	*r* = −0.41, *p* = 0.14 (linear) *r* = −0.66, *p* = 0.0102 * (logarithmic)	*r* = −0.45, *p* = 0.10 (linear)
**6296**	Terreusinol	*r* = −0.41, *p* = 0.15 (linear) *r* = −0.65, *p* = 0.012 * (logarithmic)	*r* = −0.45, *p* = 0.10 (linear)
**3152**	Amoxapine	*r* = −0.74, *p* = 0.0026 ** (linear) *r* = −0.92, *p* < 0.0001 *** (logarithmic)	*r* = −0.72, *p* = 0.0031 ** (linear)
**3073**	Elloramycin E	*r* = −0.62, *p* = 0.018 * (linear) *r* = −0.80, *p* = 0.0006 *** (logarithmic)	*r* = −0.51, *p* = 0.060 (linear)
**8925**	Sanjoinine A dialdehyde	*r* = −0.56, *p* = 0.039 * (linear) *r* = −0.76, *p* = 0.0015 ** (logarithmic)	*r* = −0.54, *p* = 0.046 (linear)
**609**	Zinolol	*r* = −0.34, *p* = 0.24 (linear) *r* = −0.59, *p* = 0.028 * (logarithmic)	*r* = −0.38, *p* = 0.18 (linear)
**2963**	Acacetin-7-glucuronosyl-(1→2)- glucuronide	*r* = −0.61, *p* = 0.020 * (linear) *r* = −0.79, *p* = 0.0009 ***(logarithmic)	*r* = −0.41, *p* = 0.14 (linear)
**3171**	Dapsone hydroxylamine	*r* = −0.39, *p* = 0.16 (linear) *r* = −0.60, *p* = 0.023 * (logarithmic)	*r* = −0.36, *p* = 0.21 (linear)
**8804**	Trapoxin A	*r* = −0.50, *p* = 0.066 (linear) *r* = −0.70, *p* = 0.0053 ** (logarithmic)	*r* = −0.54, *p* = 0.047 * (linear)
**7781**	Tricalysioside N	*r* = −0.65, *p* = 0.012 * (linear) *r* = −0.74, *p* = 0.0023 ** (logarithmic)	*r* = −0.59, *p* = 0.026 * (linear)
**9091**	Pregnadienolone-3-*O*-β-D- chacotrioside	*r* = −0.66, *p* = 0.0098 ** (linear) *r* = −0.84, *p* = 0.0102 * (logarithmic)	*r* = −0.68, *p* = 0.0079 ** (linear)
**4347**	Perphenazine	*r* = −0.52, *p* = 0.059 (linear) *r* = −0.66, *p* = 0.0002 *** (logarithmic)	*r* = −0.52, *p* = 0.056 (linear)
**3836**	Silidianin	*r* = −0.59, *p* = 0.027 * (linear) *r* = −0.72, *p* = 0.0034 ** (logarithmic)	*r* = −0.53, *p* = 0.052 (linear)
**8800**	Droperidol	*r* = −0.75, *p* = 0.0020 ** (linear)	*r* = −0.61, *p* = 0.019 * (linear)

Note: When the coefficient was better in a logarithmic model in terms of *r* than in a linear model, both are shown. If not, only the coefficient of a linear model is shown. Asterisks indicate levels of statistical significance; *: *p* < 0.05, **: *p* < 0.01, ***: *p* < 0.001.

## Data Availability

The data presented in this study and the source data are available in this article and in the Supplementary Materials.

## Supplementary Materials

The following supporting information can be downloaded at: https://www.mdpi.com/article/10.3390/life12010115/s1, Supplementary File S1: LCMS_result field data KDRI, Supplementary File S2: LCMS peak data, Supplementary File S3 (PDF file) contains Supplementary Results and Discussion (including Supplementary Figure S1) and Supplementary Table S1. Note on Supplementary File S1: To open 2D Chromatogram in the “2DView” worksheet, select either “m/z(Detected)” or “Exact Mass” at the cell next to “Vertical axis”, and select a sample at the cell next to “Group”. To open MS/MS spectrogram in the “MS2” worksheet, double-click a peak number. The “PCA” and “t-Test” worksheets are not useful due to unrelated samples in the file.
