# Supplementary material for: Metabolomic Profiles of the Creeping Wood Sorrel Oxalis corniculata in Radioactively Contaminated Fields in Fukushima: Dose-Dependent Changes in Key Metabolites
Source: Life (Basel). 2022 Jan 13;12(1):115. doi: 10.3390/life12010115 (PMC8780803; doi:10.3390/life12010115)
Supplement: Supplementary file 1 [file life-12-00115-s001.zip › Supplementary Materials Life R3 Dec 22 2021 to be submitted.pdf]

Supplementary Materials

Metabolomic Profiles of the Creeping Wood Sorrel *Oxalis corniculata* in Radioactively Contaminated Fields in Fukushima: Dose-Dependent Changes in Key Metabolites

Ko Sakauchi [1], Wataru Taira [1,2], and Joji M. Otaki [1,*]

[1] The BCPH Unit of Molecular Physiology, Department of Chemistry, Biology and Marine Science, Faculty of Science, University of the Ryukyus, Okinawa 903-0213, Japan
[2] Research Planning Office, University of the Ryukyus, Okinawa 903-0213, Japan
* Correspondence: otaki@sci.u-ryukyu.ac.jp; Tel.: +81-98-895-8557

1. Supplementary Results and Discussion (including Supplemntary Figure S1)

1.1. Correlation between Ground Dose and Radioactivity Concentration

We examined a relationship between ground radiation dose rate and radioactivity concentration of ^{137}Cs in leaf samples. They were highly correlated (Pearson correlation coefficient r = 0.81, p = 0.0004). Similar results have been obtained in the previous studies: r = 0.63, p < 0.001 [24] and ρ = 0.80, p = 0.33 [27]. These correlation coefficients were not very close to 1, suggesting that absorption and transportation of ^{137}Cs from soil to leaves may depend on several factors such as soil composition, environmental temperature, and genetic background. Additionally, human activities such as decontamination process may also influence the correlation between the two. Considering that leaves of this plant are positioned closely to the ground, the plant is likely subjected to both internal and external exposures. Ground radiation (x-axis) includes entire radiation doses from various radionuclides in addition to ^{137}Cs in the field environment, but radioactivity concentration (y-axis) includes only ^{137}Cs. Therefore, we decided to focus primarily on the ground radiation dose rate for subsequent analyses.

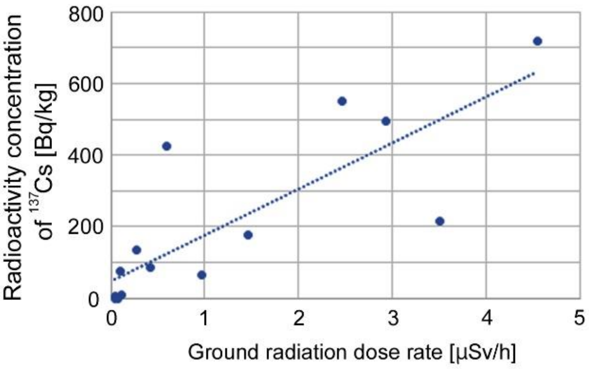

Supplementary Figure S1. Scatter plot between ground radiation dose rate [μSv/h] and radioactivity concentration of ^{137}Cs [Bq/kg] in the plant leaf samples. A linear fit model was drawn. These two factors were correlated (r = 0.81, p = 0.0004).

2. Supplementary Table

Supplementary Table S1. 93 ANOVA-positive peaks and their raw *p*-values and FDR values (FDR < 0.05)

No.	*p*	FDR	No.	*p*	FDR	No.	*p*	FDR	No.	*p*	FDR
**5658**	7.8E-9	6.2E-5	**3033**	2.9E-5	0.0090	**2384**	0.00016	0.026	**4822**	0.00045	0.049
**6246**	4.2E-8	0.00017	**8775**	2.9E-5	0.0090	**3432**	0.00017	0.027	**147**	0.00046	0.049
**5741**	1.6E-6	0.0034	**6614**	3.0E-5	0.0090	**9530**	0.00017	0.027	**8378**	0.00047	0.049
**8903**	1.8E-6	0.0034	**4472**	3.8E-5	0.011	**4762**	0.00018	0.027	**5009**	0.00048	0.049
**4969**	2.1E-6	0.0034	**4800**	4.8E-5	0.013	**8508**	0.00018	0.027	**885**	0.00049	0.049
**5636**	2.6E-6	0.0034	**6624**	5.2E-5	0.014	**3542**	0.00023	0.033	**7563**	0.00050	0.049
**4925**	3.3E-6	0.0036	**9396**	5.6E-5	0.014	**3171**	0.00024	0.035	**5348**	0.00050	0.049
**7481**	4.0E-6	0.0036	**9368**	6.2E-5	0.015	**8451**	0.00025	0.036	**4347**	0.00051	0.049
**7234**	4.2E-6	0.0036	**5402**	6.4E-5	0.015	**474**	0.00026	0.037	**6821**	0.00052	0.049
**4887**	4.6E-6	0.0036	**4261**	6.5E-5	0.015	**4345**	0.00027	0.037	**5353**	0.00052	0.049
**9321**	7.5E-6	0.0055	**4039**	7.1E-5	0.016	**3172**	0.00028	0.038	**3836**	0.00052	0.049
**832**	9.3E-6	0.0056	**4388**	7.7E-5	0.017	**9211**	0.00029	0.039	**7435**	0.00054	0.049
**7156**	9.4E-6	0.0057	**8925**	7.8E-5	0.017	**8804**	0.00030	0.039	**7256**	0.00054	0.049
**3180**	1.0E-5	0.0058	**2049**	8.2E-5	0.017	**4702**	0.00031	0.040	**9372**	0.00054	0.049
**7764**	1.1E-5	0.0059	**4943**	9.4E-5	0.019	**2**	0.00031	0.040	**178**	0.00055	0.049
**6296**	1.7E-5	0.0081	**5464**	9.4E-5	0.019	**9338**	0.00034	0.042	**3038**	0.00055	0.049
**3152**	1.7E-5	0.0081	**1856**	0.00010	0.019	**7781**	0.00034	0.042	**750**	0.00055	0.049
**1197**	2.2E-5	0.0090	**609**	0.00011	0.021	**7968**	0.00035	0.042	**8800**	0.00056	0.049
**3073**	2.4E-5	0.0090	**2963**	0.00011	0.021	**4578**	0.00037	0.044	**8933**	0.00057	0.049
**7155**	2.4E-5	0.0090	**1109**	0.00012	0.021	**3204**	0.00038	0.045	**7719**	0.00057	0.049
**4880**	2.6E-5	0.0090	**9322**	0.00012	0.021	**9091**	0.00040	0.046	**2562**	0.00057	0.049
**9023**	2.7E-5	0.0090	**4745**	0.00013	0.022	**9335**	0.00041	0.047			
**3404**	2.9E-5	0.0090	**5617**	0.00013	0.023	**6930**	0.00043	0.047			
**6347**	2.9E-5	0.0090	**4912**	0.00014	0.024	**8935**	0.00043	0.047			

Note: Singularly annotated upregulated and downregulated peaks are shown in red and blue, respectively.
